# Achilles tendon compliance influences tendon loading more than Achilles tendon twist in Achilles tendinopathy: a musculoskeletal modeling approach

**DOI:** 10.3389/fbioe.2024.1399611

**Published:** 2024-07-18

**Authors:** Ine Mylle, Alessia Funaro, Marion Crouzier, Stijn Bogaerts, Benedicte Vanwanseele

**Affiliations:** ^1^ Human Movement Biomechanics Research Group, Department of Movement Science, KU Leuven, Leuven, Belgium; ^2^ Movement Interactions Performance, MIP, UR 4334, Nantes University, Nantes, France; ^3^ Locomotor and Neurological Disorders Research Group, Department of Development and Regeneration, KU Leuven, Leuven, Belgium; ^4^ Department of Physical and Rehabilitation Medicine, University Hospitals Leuven, Leuven, Belgium

**Keywords:** Achilles tendinopathy, musculoskeletal modeling, twist, compliance, triceps surae

## Abstract

The Achilles tendon exhibits anatomical variations in subtendon twist among individuals, and its compliance can change due to conditions like Achilles tendinopathy. However, current musculoskeletal models overlook these material and morphological variations. This study aimed to investigate the impact of altering Achilles subtendon insertion points and compliance on the triceps surae muscle forces, and therefore tendon loading, during dynamic exercises in one Achilles tendinopathy patient. First, subtendon insertion points were altered in the musculoskeletal model based on a subject-specific 3D freehand ultrasound model and for three types of subtendon twists: low, medium, and high. Second, tendon compliance was modeled based on experimental values, creating three musculoskeletal models: compliant, mean, and stiff. Results indicated that tendon compliance had a larger effect than tendon twist on triceps surae muscle forces. Altering subtendon insertion points to the three types of twist showed a maximal change of 2.3% in muscle force contribution compared to the no-twist model. During the eccentric rehabilitation exercise—a common exercise choice during rehabilitation—the compliant tendon model showed substantial differences compared to the generic (control) musculoskeletal model, resulting in decreased gastrocnemius medialis (−3.5%) and gastrocnemius lateralis (−3.2%) contributions and increased soleus contribution (+ 6.6%). Our study results highlight the necessity of incorporating tendon compliance in musculoskeletal models to accurately predict triceps surae muscle forces, especially in individuals with increased tendon compliance, such as patients with Achilles tendinopathy. Such findings contribute to more accurate predictions of muscle forces and hence, personalized rehabilitation strategies.

## 1 Introduction

The Achilles tendon is mechanically loaded through the triceps surae muscle forces—soleus (SOL), the gastrocnemius medialis (GM), and the gastrocnemius lateralis (GL) (Kharazi et al., 2021). As the Achilles tendon is mechanosensitive and adapts to mechanical loading (Magnusson et al., 2010; Joseph et al., 2014), the quantification of the triceps surae muscle forces is crucial to better understand (mal) the adaptation of the tendon. Currently, these muscle forces cannot be directly measured non-invasively. Different methods are used to estimate Achilles tendon load, such as an inverse dynamics approach; for a complete overview of different methods, see Finni and Vanwanseele (2023); however, they only provided a global estimate of the total Achilles tendon load. As the Achilles tendon is composed of three subtendons, each originating from its respective triceps surae muscle, the load on the tendon is not uniform (Mylle et al., 2023). Hence, a good estimation of the individual muscle forces when loading the Achilles tendon is crucial. Musculoskeletal modeling has emerged as a useful tool in biomechanics research, providing and enabling a deeper understanding of human movement and, hence, gaining insights into the loading of musculoskeletal tissues (De Groote and Falisse, 2021). For example, with the use of musculoskeletal modeling simulations, the muscle forces can be measured through a dynamic optimization approach (De Groote et al., 2016). However, these models still remain a simplification of the human body as the insertion of the muscle–tendon actuators and tendon compliance are generic and do not consider differences in morphological and/or material properties.

The three subtendons exhibit some degrees of twist from proximal to distal, caused by the collagen fiber arrangement (Pękala et al., 2017). This twist leads to a helical structure, which might enhance force transmission along the tendon during movement due to its effective design for energy storage and release (Bojsen-Møller and Magnusson, 2019) and affect the strain distribution within the tendon (Shim et al., 2018). In healthy tendons, the twist contributes to the ability to withstand tensile loads; hence, it has been hypothesized that tendon twist is linked to the development or progression of Achilles tendinopathy, as it might impact the mechanical behavior of Achilles tendon (Edama et al., 2016; 2019). This tendon twist not only redistributes the load and strain within the tendon but also influences the contribution of forces of each of the triceps surae muscle to the plantar flexor torque due to the altered insertion point on the calcaneus and hence displays differences in the individual moment arm of each triceps surae muscle (Rasske et al., 2017). By changing the insertion points of the muscle–tendon actuators, musculoskeletal models can help understand how tendon twist would influence the contribution of triceps surae muscle forces.

In the case of Achilles tendinopathy patients, the compliance of the Achilles tendon is increased when compared to asymptomatic participants (Arya and Kulig, 2010). Compliant tendons are known to reduce muscle fiber contraction velocities (Lichtwark and Barclay, 2010) or increase change in length (Cox et al., 2019). As the capacity for generating muscle force is affected by both muscle lengths and velocities (Arnold et al., 2013), these changes will influence the muscle force generation and therefore the load on the tendon. It is therefore possible that in the case of tendinopathy, muscle contractile behavior is altered and impacts the load borne by the tendon. Again, the use of musculoskeletal models could provide insights into how changes in tendon compliance may influence the muscle distribution.

Tendon twist and compliance are two important factors that could lead to variations in the distribution of force between the gastrocnemius and soleus muscles, leading to difference in load on the Achilles tendon. Understanding the role of tendon twist and compliance on the triceps surae muscle force distribution can provide crucial insights into the loading of the Achilles tendon in Achilles tendinopathy. To our knowledge, no previous study has yet investigated the influence of the different insertion points of the subtendon or different degrees of tendon compliance on the triceps surae muscle forces in patients with Achilles tendinopathy using musculoskeletal modeling. This study investigates how subtendon insertion points and compliance influence triceps surae muscle forces of individuals with Achilles tendinopathy, by employing a musculoskeletal modeling approach. Specifically, it compares models with varying degrees of tendon twist and compliance and assesses their deviations based on the standard generic model.

## 2 Materials and methods

### 2.1 Participant

One participant (male, 47 y, 184 cm, 85 kg) with clinically diagnosed Achilles tendinopathy (VISA-A: 65), selected from a larger cohort, volunteered to participate in this study and gave written informed consent. This study was approved by the local Ethical Committee KU/UZ Leuven (S63532). The participant was screened by a medical doctor in order to verify the following inclusion and exclusion criteria: i) having a documented history of recurring pain in the Achilles tendon lasting for over 6 consecutive weeks, along with episodes of worsening and improvement within the last 5 years, ii) experiencing pain upon palpation originating from the mid-portion of the Achilles tendon, iii) confirmation of Achilles tendinopathy by imaging, i.e., the presence of focal thickening and hypoechoic areas, and iv) no (previous) injuries to the ankle/foot complex or the Achilles tendon and/or a systemic disease affecting the collagenous tissue.

### 2.2 Experimental design

Upon arrival in the laboratory, the participant was given the Victorian Institute of Sport Assessment—Achilles questionnaire (VISA-A)—to quantify pathology severity and the International Physical Activity Questionnaire (IPAQ) to assess physical activity. Upon completion of a standardized warm-up, three-dimensional freehand ultrasonography (3DfUS) was conducted to measure the morphological and mechanical properties of the Achilles tendon during rest. Thereafter, the participant was prepared to perform three different exercises barefoot in a randomized order: a walk, an eccentric bilateral heel drop, and a concentric bilateral heel rise in the movement laboratory. These exercises were chosen based on the Alfredson eccentric protocol (Alfredson et al., 1998). For each exercise, three trials were recorded upon visual and verbal guidance by the researcher prior to and during the exercise performance, following the familiarization trial. The bilateral heel-rise and heel-drop exercises were performed in a standardized way on a 15-cm box placed on the force plate for a total duration of 3 s and controlled with a metronome set at 1 Hz. Movement was instructed to reach complete plantar and dorsiflexion within their capabilities. Additionally, the patient’s foot progression angle was controlled by a tape line on the box to ensure a neutral (0°) angle. Walking was instructed to be done at a self-selected and comfortable pace on the straight walkway, without specifying the location of the embedded force plate, so that gait kinematics were not altered. The tendinopathy leg was placed onto the (box placed on top) embedded force plate for all three exercises.

### 2.3 3D freehand ultrasonography (3DfUS)

3DfUS is a previously validated technique (Obst et al., 2014) used to capture and create 3D reconstructions of the Achilles tendon *in vivo* by combining 2D B-mode ultrasonography (ArtUS, UAB Telemed, Vilnius, Lithuania) with the 3D motion capture system (OptiTrack, NaturalPoint Inc., Corvallis, OR, United States) in the 3D slicer software (open source: www.slicer.org, version 4.11; (Fedorov et al., 2012). The Achilles tendon was imaged from the calcaneal insertion to the GM muscle–tendon junction in the transverse plane at rest and during submaximal plantarflexion contractions. Participants were lying prone—extended hip and knee joint—on an isokinetic dynamometer (Biodex system 4 MVP, Biodex Medical Systems, New York, United States). The participant’s most affected ankle was fixated and strapped against a footplate in a neutral ankle angle—foot perpendicular to the shank—with the lateral malleolus carefully aligned with the ankle axis of rotation. The free Achilles tendon length—from calcaneal insertion to the SOL muscle–tendon junction—and 3D volume were segmented and calculated during rest and each submaximal contraction (Funaro et al., 2022).

### 2.4 Musculoskeletal modeling

#### 2.4.1 Generic model

The generic gait2392 musculoskeletal model (Delp et al., 1990) was modified by removing the degrees of freedom in the lumbar joint and adding a degree of freedom in the knee joint (varus–valgus). The model finally consisted of 43 lower limb Hill-type muscles and 22 degrees of freedom. This generic model will be considered the healthy control subject.

#### 2.4.2 Tendon twist model

**FIGURE 1:**
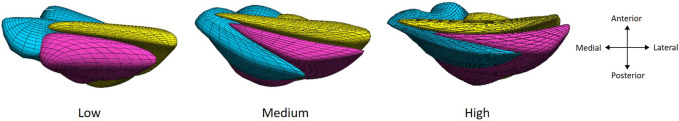
Three models of increasing tendon twisting (from left to right) based on a finite element modeling approach with the three subtendons: soleus (bleu), gastrocnemius medialis (pink), and gastrocnemius lateralis (yellow), displayed from the calcaneal insertion for a right leg.

To add subtendon insertion points into the musculoskeletal model, adjustments to the generic musculoskeletal model were made in OpenSim 3.3 (OpenSim, Stanford, United States) to account for the differences in subtendon insertion points of the individual muscle–tendon actuators. Based on the patient’s 3D free Achilles tendon reconstruction, a free Achilles tendon finite element mesh was constructed using Materialise 3-matic (Materialise NV, Leuven, Belgium) (Funaro et al., 2022). The tendon model was divided into three subtendons based on the geometrical description, while also applying twist angles to produce three twisted structures, with both features corresponding to the AT twist classification described by Pękala et al. (2017). As such, three different tendon twist musculoskeletal models were created (Figure 1). The midpoint of each subtendon at the calcaneal insertion was retrieved from the finite element model, as well as the Achilles tendon midpoint, to align its coordinate system to the coordinate system of the musculoskeletal modeling. Insertion points of the three triceps surae muscle–tendon actuators were then adjusted from the generic musculoskeletal model based on the anatomical location of each subtendon’s midpoint to create the three different types of tendon twists. Additionally, since tendon shapes are very subject-specific (Szaro et al., 2009; Edama et al., 2015; Pękala et al., 2017; Yin et al., 2021), insertion points of each muscle–tendon actuator were moved by factors of 50% and 100% of its original distance from the Achilles tendon midpoint to represent larger tendons. Final locations of the insertion points for all different tendon twist models compared to the generic model can be found in Supplementary Figure S1.

#### 2.4.3 Tendon compliance model

Achilles tendon compliance values were experimentally measured (Arya and Kulig, 2010) and were converted to the normalized tendon stiffness. The generic musculoskeletal model assumes a normalized tendon stiffness value of 35 for all Hill-type muscles (Zajac, 1989). Therefore, we considered a normalized tendon stiffness value of 35 for our generic model so that it is equal to the mean experimental value measured (Arya and Kulig, 2010). In their study, considering that the AT group had a mean stiffness value that was 20% lower, a normalized value of 28 for the mean (AT) model was observed. This normalized value for the mean model was then used to calculate the value of the most compliant model based on mean and 2 standard deviations to acquire a value of 21. While for the stiffest model, the mean and 2 standard deviations of the control group obtained a value of 47.

### 2.5 Data analysis

An extended Plug-In Gait marker set, composed of 34 retroreflective markers, was placed on the participant’s lower body while capturing 3D marker trajectories through 10 infrared motion capturing cameras (Vicon, Oxford Metrics, Oxford, United Kingdom) with a sampling rate of 100 Hz and ground reaction forces embedded in the walkway (AMTI Inc., MA, United States) and sampled at 1,000 Hz. Upon scaling the musculoskeletal model according to the subject’s characteristics obtained during a static trial, joint angles were calculated through a Kalman smoothing algorithm (De Groote et al., 2008), while joint moments were calculated through an inverse dynamic approach based on the joint angle and ground reaction force data in OpenSim 3.3 (OpenSim, Stanford, United States). The data were low-pass filtered with a 6 Hz cutoff frequency. Through a dynamic optimization method (De Groote et al., 2016), in which the muscle redundancy problem was solved through the minimization of the sum of squared muscle activation, triceps surae muscle (GM, GL, and SOL) forces were calculated. A schematic overview of the modeling workflow can be found in Supplementary Figure S2. Muscle force-sharing strategies were calculated for each exercise by dividing the individual muscle force by the sum of the three muscle forces at the moment of peak triceps surae force. All variables were normalized to the duration of the exercise. For the walking exercise, ground contact times for initial contact (0%) and toe-off (100%) were determined based on the ground reaction force data. The lowest and highest heel marker positions were used to determine the start (0%) and end (100%) of the bilateral heel-rise and heel-drop exercises.

## 3 Results

### 3.1 Tendon twist

Tendon twist had a minimal influence on the triceps surae muscle forces at peak triceps surae force, as the greatest difference in muscle contribution was a 2.3% change, compared to the generic model, for all exercises performed.

#### 3.1.1 Bilateral heel drop

**FIGURE 2:**
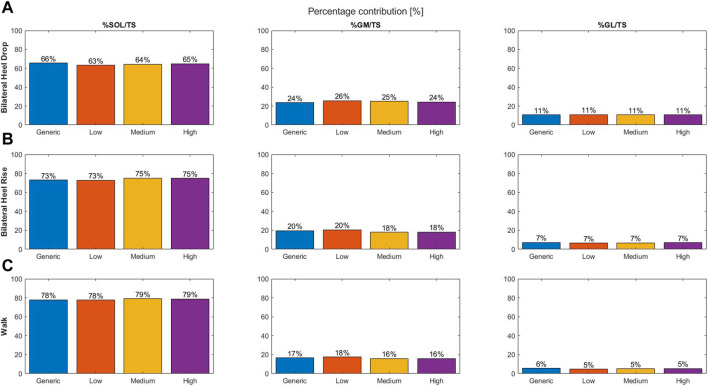
Percentage contribution of the individual muscle force to the total triceps surae muscle force at the moment of peak triceps surae force for different types of tendon twist models: generic (no twist; blue), low twist (orange), medium twist (yellow), and high twist (purple), and for the three different dynamic exercises: bilateral heel drop **(A)**, bilateral heel rise **(B)**, and walk **(C)**.

The SOL muscle force and its contribution to the total triceps surae muscle force increased with increase in degrees of twisting, while the opposite was true for the GM muscle force and contribution (Figure 2A). The generic model, with no tendon twist, displayed the lowest contribution for the GM and GL and the largest contribution for SOL.

#### 3.1.2 Bilateral heel rise

Increasing twist resulted in a tendency toward increased SOL and GL and decreased GM muscle force and contribution (Figure 2B). The generic model showed the largest GL and GM and lowest SOL contribution compared to the three different models of tendon twist.

#### 3.1.3 Walking

A tendency toward increased GL muscle force and contribution was observed with increasing twist (Figure 2C). Medium- and high-twist models do not display differences for the GM and SOL contributions; however, low twist models show a decrease in SOL and increase in GM contribution. The generic model’s muscle contribution fits well between low twist and medium/high twist for the SOL and GM, but is the largest for the GL.

#### 3.1.4 Effect on the moment arm

In addition, in larger tendons, where insertion points of the muscle–tendon actuator were further away from the origin (50% or 100% increase), the difference in triceps surae muscle force-sharing remained small. Even with changes of up to 2 mm (SOL), 1 mm (GM), and 2 mm (GL) in the moment arm of the muscle–tendon actuator compared to the generic model, the largest differences were observed for the GM compared to the generic model, with differences up to 3.5%, 6.4%, and 5.5% for the bilateral heel drop, bilateral heel rise, and walk, respectively (Supplementary Figure S1).

### 3.2 Tendon compliance

Increasing tendon compliance had an influence on the triceps surae muscle forces at peak triceps surae forces and generated differences in muscle contribution of up to 6.6% compared to the generic model.

#### 3.2.1 Bilateral heel drop

**FIGURE 3:**
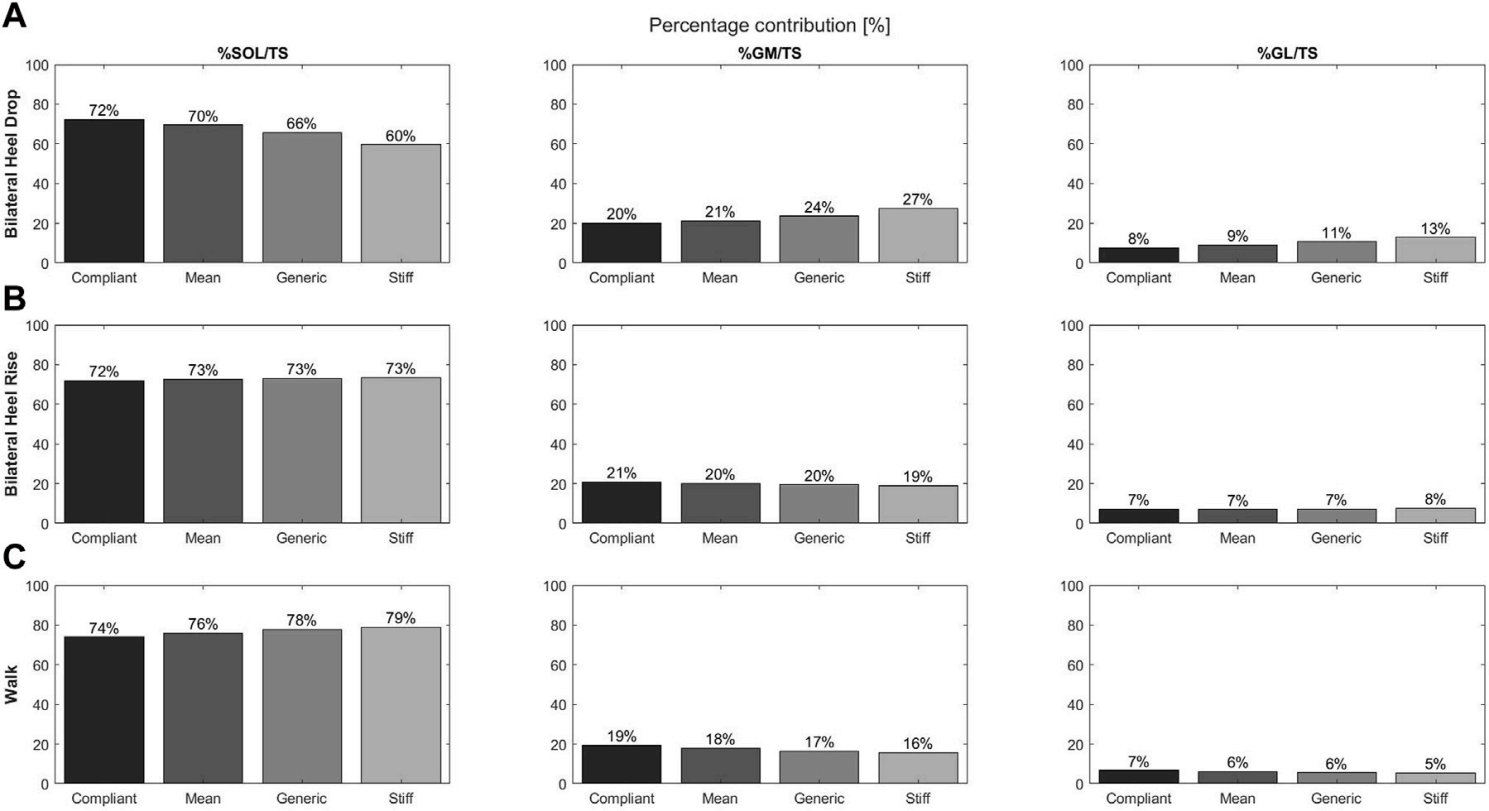
Contributions of individual muscle force to the total triceps surae muscle force for the soleus, gastrocnemius medialis, and gastrocnemius lateralis and during the bilateral heel drop **(A)**, bilateral heel rise **(B)**, and walk **(C)** at the moment of peak triceps surae force for the four tendon compliance models: compliant (21; black), mean (28; dark gray), generic (35; concrete), and stiff (47; light gray).

With increasing compliance, SOL muscle force increased, while GM and GL muscle forces decreased (Figure 3A). Differences of + 12.6%, −7.3%, and −5.4% were found for the SOL, GM, and GL contributions, respectively, when comparing the compliant model to the stiff model, while differences of + 6.6%, −3.5%, and −3.1% were found in comparison to the generic model, respectively.

#### 3.2.2 Bilateral heel rise

Differences in muscle contribution with increasing tendon compliance were less obvious; however, the compliant model displayed the largest differences with the SOL showing decreased contributions (−1.2%), while GM contribution increased by 1.2% compared to the generic model (Figure 3B).

#### 3.2.3 Walking

SOL muscle force and contributions to the total triceps surae muscle force decreased with increasing compliance, while GM and GL forces and contributions had the opposite effect (Figure 3C). In comparison to the generic model, the differences were larger for the compliant model than for the stiffest model.

### 3.3 Tendon twist x compliance

A cross-analysis of both tendon twisting and tendon compliance revealed similar results, with tendon compliance having the largest effect on the triceps surae muscle force-sharing behavior, independent of the type of tendon twisting. For the bilateral heel-drop exercise, SOL showed an increased contribution and GM and GL showed a decreased contribution with increasing compliance and increasing twist (Table 1). Similarly, during both the bilateral heel-rise and walking exercises, all compliance models displayed a tendency toward increased SOL and GL contributions but decreased GM with increasing twist—medium and high twist are fairly similar for the SOL and GM. However, with increasing compliance, SOL contribution decreased, while the GM contribution increased.

**TABLE 1:** Contributions of the individual triceps surae muscle force to the total triceps surae force at the moment of peak triceps surae muscle force: a cross-sectional analysis of tendon twist types and tendon compliance models compared to the generic model with no twist induced.

Cross-sectional analysis twist/compliance		% SOL/TS			% GM/TS			% GL/TS		
		Low	Medium	High	Low	Medium	High	Low	Medium	High
Bilateral heel drop	Generic_no twist	65, 63			23, 65			10, 72		
	Compliant	70, 74	71, 73	71, 51	21, 62	20, 73	20, 93	7, 64	7, 53	7, 56
	Mean	67, 44	68, 57	68, 95	23, 23	22, 24	21, 87	9, 33	9, 19	9, 17
	Generic	63, 32	64, 29	64, 90	25, 79	24, 95	24, 35	10, 89	10 ,76	10, 74
	Stiff	57, 14	57, 78	58, 49	29, 80	29, 22	28, 44	13, 07	13 ,00	13, 01
Bilateral heel rise	Generic_no twist	73, 14			19, 63			7, 23		
	Compliant	71, 97	73, 14	72, 56	21, 55	20, 12	20, 42	6, 48	6, 74	7, 02
	Mean	72, 55	74, 49	73, 99	21, 04	18, 87	19, 15	6, 41	6, 64	6, 86
	Generic	72, 90	75, 21	74, 80	20, 49	18, 01	18, 24	6, 60	6, 78	6, 96
	Stiff	72, 89	75, 32	75, 09	20, 05	17, 52	17, 62	7, 06	7, 16	7, 29
Walk	Generic_no twist	77, 81			16, 54			5, 65		
	Compliant	74, 32	74, 61	74, 11	19, 73	19, 14	19, 37	5, 95	6, 25	6, 52
	Mean	75, 92	76, 96	76, 42	18, 88	17, 54	17, 83	5, 20	5, 49	5, 76
	Generic	77, 67	79, 08	78, 72	17, 59	15, 83	15, 97	4, 74	5, 09	5, 31
	Stiff	78, 96	80, 36	80, 04	16, 33	14, 75	14, 93	4, 71	4, 89	5, 03

## 4 Discussion

This simulation study provides insights into the influence of altering subtendon insertion points associated with tendon twist and altering tendon compliance on the muscle forces during different dynamic exercises. Findings suggest that tendon compliance has a larger influence on the triceps surae muscle forces than the degree of tendon twist. Specifically, during the commonly performed eccentric rehabilitation exercises, large differences were observed between the compliant tendon and the generic model.

Our results revealed that modeling a more compliant tendon leads to different triceps surae forces and contributions compared to modeling stiffer tendons. GM and GL contributions decreased, while the SOL contribution to total triceps surae force increased during the eccentric bilateral heel-drop exercise with a compliant tendon. In other words, a more compliant tendon might require an increased involvement of the SOL during eccentric contractions, yet the reverse effect where increased contribution leads to greater compliance might exist, but is not yet understood. Indeed, differential muscle forces within the different triceps surae muscles were found during different dynamic exercises (Mylle et al., 2023). This was also demonstrated with the use of finite element modeling, where individual muscle contributions of 63% for SOL, 23% for GM, and 14% for GL were found during eccentric contractions in healthy participants (Handsfield et al., 2017), which aligns well with our results for the generic model (66% SOL, 24% GM, and 11% GL). Additionally, Arnold et al. (2013) found that differences in muscle dynamics occur in different triceps surae muscles due to changes in fiber length and contraction velocities. Furthermore, force generation is dependent on tendon compliance as normal fiber length will be affected (Zajac, 1989). Hence, it was expected that differences exist for the different muscles and compliance models. In case of a compliant tendon, the muscle will function at shorter lengths and reduced fiber contraction velocities during a cyclic contraction (Lichtwark and Barclay, 2010). With increasing compliance models, a shift on the force–length curve toward shorter fascicle lengths was observed for all muscles and in all exercises (Supplementary Figure S3). During the eccentric bilateral heel-drop exercise, fascicle lengths operate on the descending limb of the curve, and hence, more optimal lengths for force production are achieved in the compliant model. Passive forces in the GM and GL may play a role in stiffer models to compensate for its decreased force–length potential. The opposite trend was observed during the concentric movements: the bilateral heel rise and the walk, where less optimal fascicle lengths and a lower contribution in the SOL were achieved. GM and GL operate on the plateau region, and as a result, smaller differences in force contribution were found compared to stiffer models. Increased compliance also resulted in reduced contraction velocities (Supplementary Figure S4); however, near isometric contractions were performed, and muscle force generating capacity was not impacted, in comparison to stiffer models.

Considering that Achilles tendinopathy patients have a more compliant tendon, it is worthwhile to model the tendon compliance when investigating the triceps surae muscle force-sharing strategies. When compared to healthy participants, patients with Achilles tendinopathy demonstrated an altered triceps surae force-sharing behavior, with a significantly increased SOL and a trend toward decreased GM and GL force during the bilateral heel-drop exercise (Mylle et al., 2023). Our current study results confirmed this finding; for this same exercise, the compliant (tendinopathy) model showed a decrease in GM (−3.48%) and GL contribution (−3.15%) and an increase in SOL contribution (+ 6.63%) compared to the generic (healthy) model. Additionally, during isometric contractions, a reduced GL contribution and activation (Crouzier et al., 2020), a reduced GL motor unit neural discharge rate (Fernandes et al., 2023), and a tendency toward reduced non-uniform intratendinous sliding in patients with Achilles tendinopathy (Couppé et al., 2020) were reported. These observations all highlight the importance of GM and GL contributions in the rehabilitation of Achilles tendinopathy patients. Therefore, future studies should include tendon compliance in simulations in which musculoskeletal modeling is used to investigate contributions of triceps surae forces.

In this musculoskeletal modeling study, increasing the tendon twist—by adjusting the location of the subtendon’s insertion point—did not have an impact on the triceps surae forces. Even when increasing the subtendon’s insertion point by a factor of 100% to represent larger tendons, muscle force contributions did not change much compared to the those of the generic model. However, it is important to note that across different models and exercises, the maximum difference in the moment arm of the individual muscle–tendon actuator was 2 mm. Even though the moment arm has a considerable influence on the magnitude of the muscle forces to achieve the experimentally observed joint torques (Rasske et al., 2017; Holzer et al., 2020), tendon twist is responsible only for changes in the subtendon moment arm, and not for the Achilles tendon moment arm. Hence, in the current musculoskeletal modeling framework, incorporating tendon twisting did not significantly affect total triceps surae force-sharing. Nevertheless, the biomechanical significance of tendon twisting cannot be neglected since tendon twisting promotes strain distributions and, hence, force production (Dean et al., 2007).

Some limitations need to be discussed. First, the data presented above are based on observations from a single subject. Achilles (sub) tendon properties are highly individualized (Edama et al., 2015; Pękala et al., 2017), leading to different responses to loading (Shim et al., 2019) across the entire AT population. In this simulation study, kinematic and kinetic data from one patient were used as input for the musculoskeletal model. To represent morphological and altered material properties known to occur in AT patients, we simulated the effect of tendon twist, larger cross-sectional areas, and tendon compliance on the muscle force-sharing behavior. Our study demonstrates the importance of including tendon compliance when investigating muscle-force sharing behavior in this specific population. Using non-invasive imaging methods to assess tendon properties, such as tendon compliance, the diagnosis of tendon injuries could be enhanced and rehabilitation can be improved (Fouré, 2016). Therefore, measurement of tendon compliance could be incorporated into standard operating procedures during clinical assessments at the time of diagnosis as a determination of impairment. This measurement could then be easily integrated into data processing related to various exercises to assess weaknesses in SOL/GM, facilitating individualized and targeted training. If this complete workflow is not feasible, measuring tendon compliance during clinical assessments can still provide valuable reference guidelines for health professionals. These guidelines can help adapt patient care strategies, such as focusing more on strengthening the GM or GL muscles during eccentric exercises by adjusting foot positioning. In addition, the effect of tendon compliance (+ 6.6%) might not be significantly different compared to the effect of tendon twist (+ 2.3%) when compared to the generic model; hence, future studies are needed to confirm the clinical relevance of these differences in a broader AT population. Second, a simple generic musculoskeletal model with individual and independent muscle–tendon actuators was used. As a result, no sliding between different actuators, and thus subtendons, was incorporated, even though sliding between Achilles subtendons is known to occur (Bogaerts et al., 2017; Clark and Franz, 2018; Couppé et al., 2020). Third, Achilles tendon twisting was induced in this model by moving the calcaneal insertion point of each muscle–tendon actuator based on the experimentally retrieved 3D model implemented in the FE model consisting of subtendons (Funaro et al., 2022). However, future modeling investigations could implement insertion points in the muscle–tendon actuator at the location of each muscle–tendon unit to represent the tendon twisting better physiologically in the musculoskeletal model at the level of the tendon. Additionally, accurate muscle force estimations are important to be used as boundary conditions in FE models (Yamamura et al., 2014; Funaro et al., 2022). Finally, estimations of triceps surae forces are based on a dynamic optimization method, which is a simulation study. Thus far, it remains unknown how individual muscle forces can be estimated non-invasively *in vivo*.

In summary, the results of this simulation study highlight the significant influence of Achilles tendon compliance on triceps surae forces and their behavior during rehabilitation exercises and walking. This developed musculoskeletal modeling workflow is important, specifically to accurately predict muscle forces in the triceps surae muscles based on individualized material properties of the tendon. Understanding these effects can guide the development of more effective and personalized rehabilitation strategies for individuals with varying tendon compliance, such as during rehabilitation from Achilles tendinopathy. Further research in this area may help refine rehabilitation protocols and improve patient outcomes.

## Data Availability

The raw data supporting the conclusions of this article will be made available by the author, without undue reservation.
