# Supplementary figures and images for: Achilles tendon compliance influences tendon loading more than Achilles tendon twist in Achilles tendinopathy: a musculoskeletal modeling approach
Source: Front Bioeng Biotechnol. 2024 Jul 18;12:1399611. doi: 10.3389/fbioe.2024.1399611 (PMC11291231; doi:10.3389/fbioe.2024.1399611)

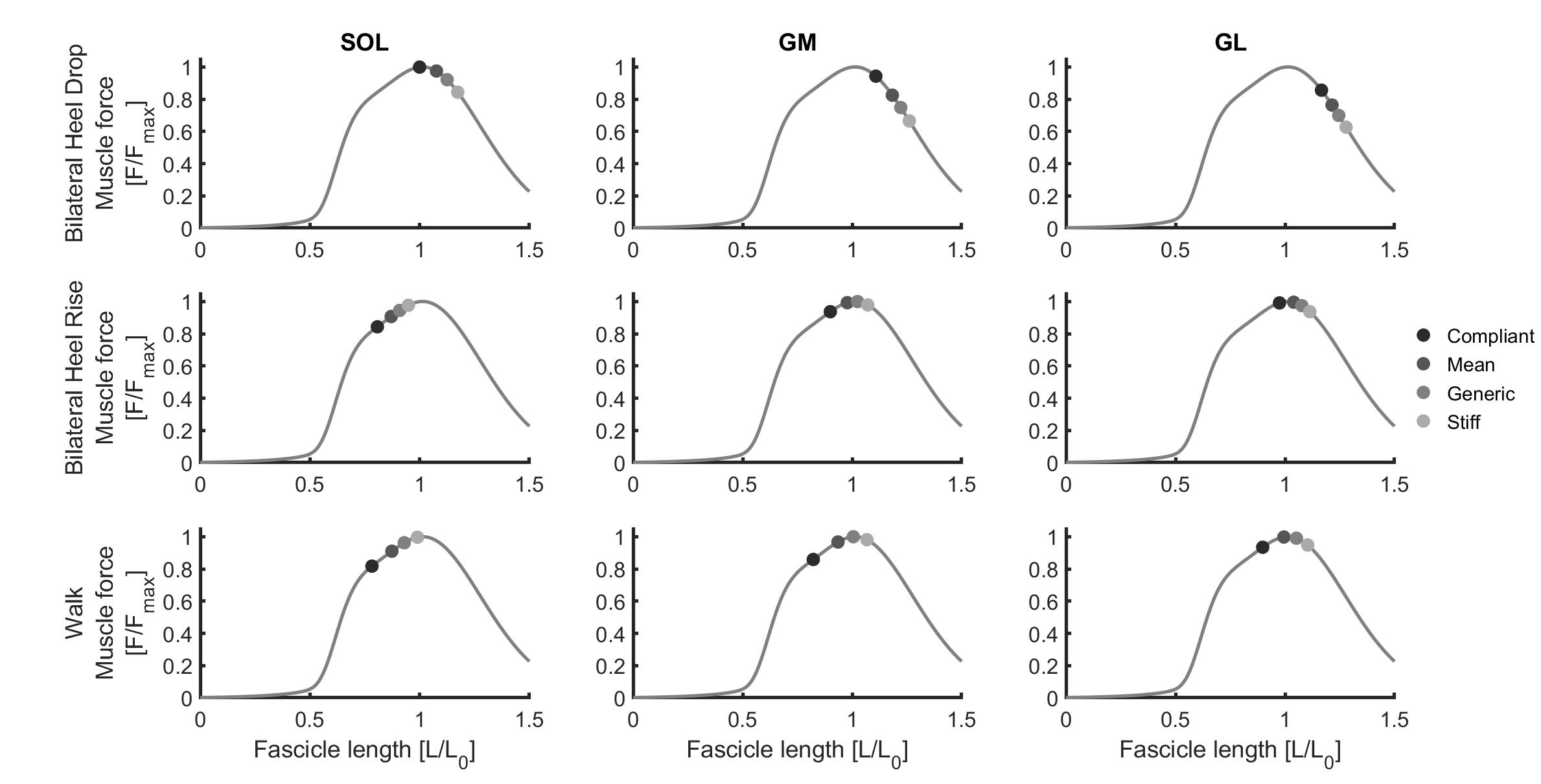

Supplement: Supplementary file 1 [file Image3.JPEG]

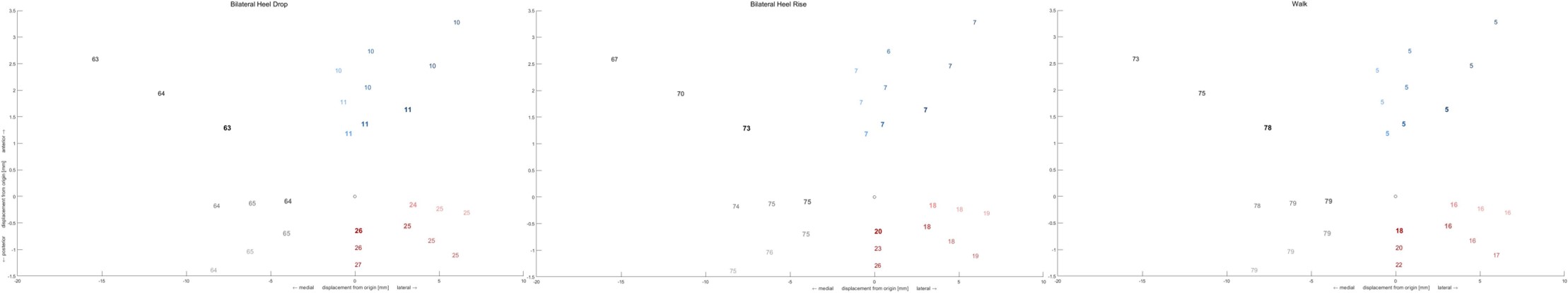

Supplement: Supplementary file 2 [file Image1.JPEG]

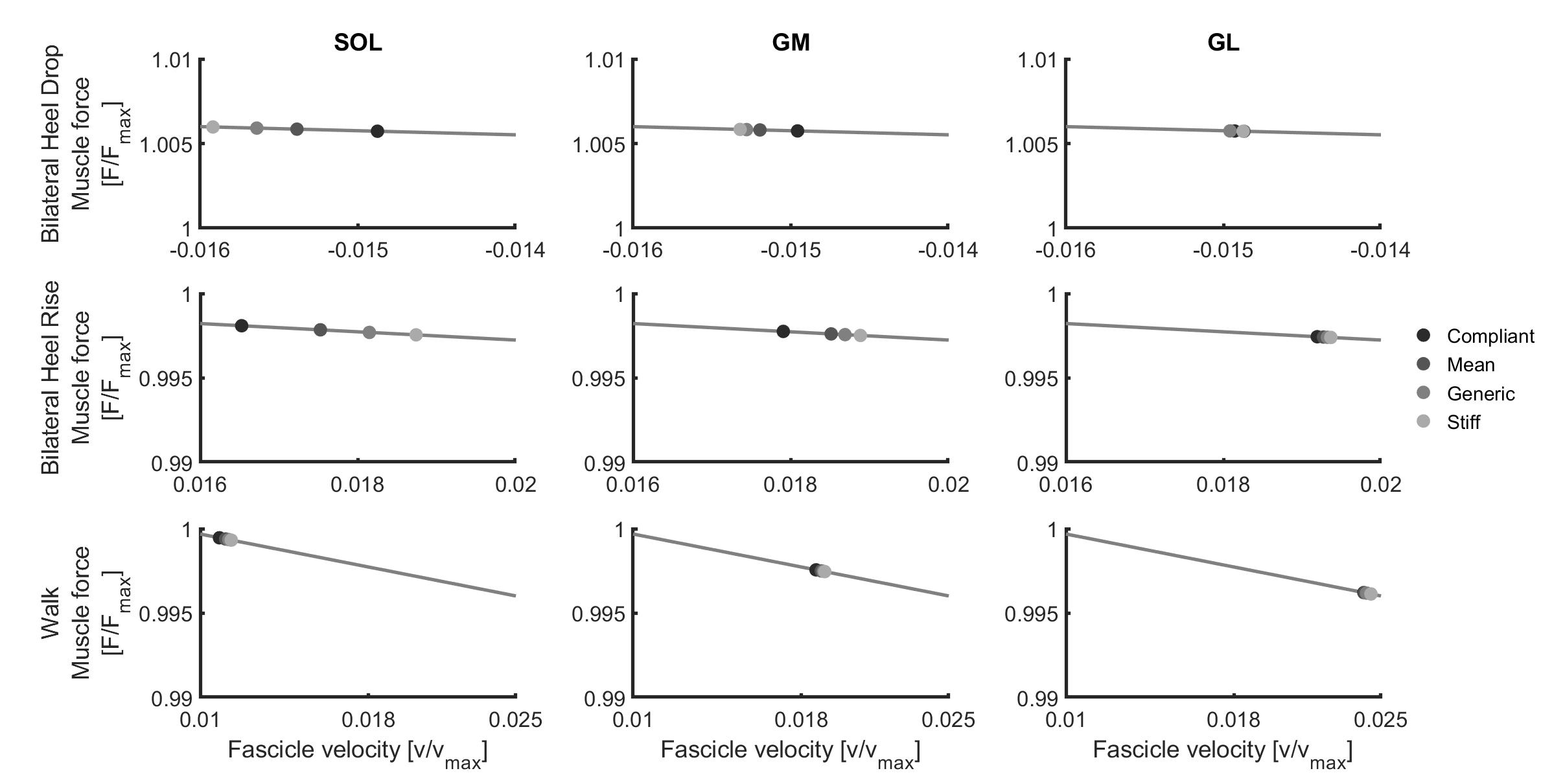

Supplement: Supplementary file 3 [file Image4.JPEG]

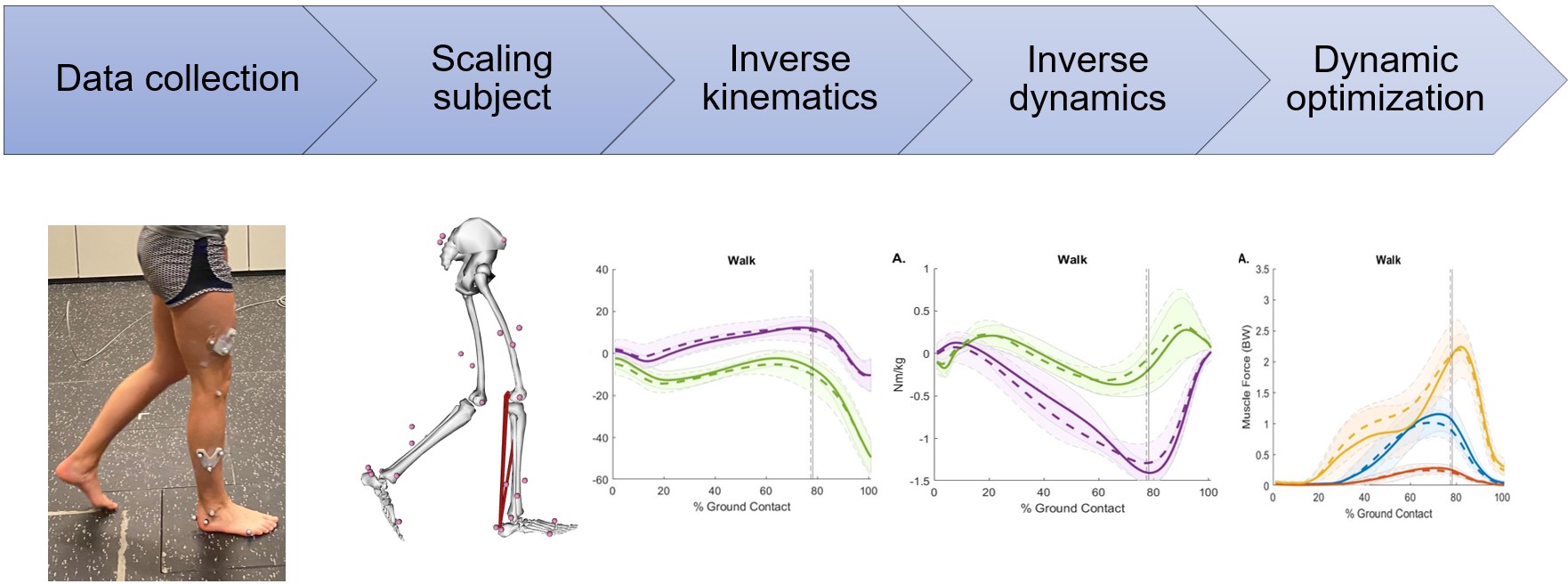

Supplement: Supplementary file 4 [file Image2.JPEG]
