# Supplementary material for: Achilles tendon compliance influences tendon loading more than Achilles tendon twist in Achilles tendinopathy: a musculoskeletal modeling approach
Source: Front Bioeng Biotechnol. 2024 Jul 18;12:1399611. doi: 10.3389/fbioe.2024.1399611 (PMC11291231; doi:10.3389/fbioe.2024.1399611)
Supplement: Supplementary file 5 [file DataSheet1.docx]

Supplementary Material

# Supplementary Figures and Tables

## Supplementary Figures


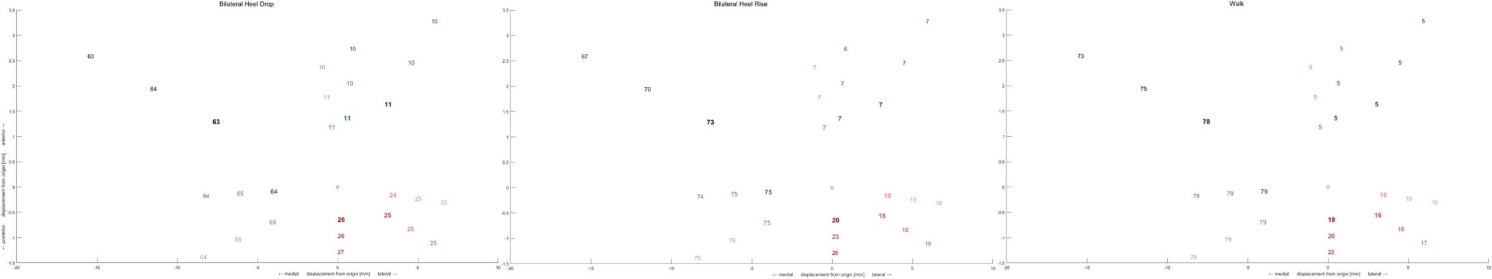


**Supplementary Figure 1**. Modelled insertion points for the three different types of twist (Type I: dark color, Type II: medium color, Type III: light color) and three different distances (generic: bold and largest font, +50%: smaller font, and +100%: smallest font) for the different subtendons (SOL: grey, GM: red, GL: blue) with its corresponding contributions to the total triceps surae force (numbers in %) during the three different exercises. The black circle represents the generic model with no twist induced. Insertion points represented here are based on the finite element model of the subject’s right leg (see Figure 1). Distance from origin (0,0) is represented in mm in the medio-lateral direction (x-axis) and the antero-posterior direction (y-axis).


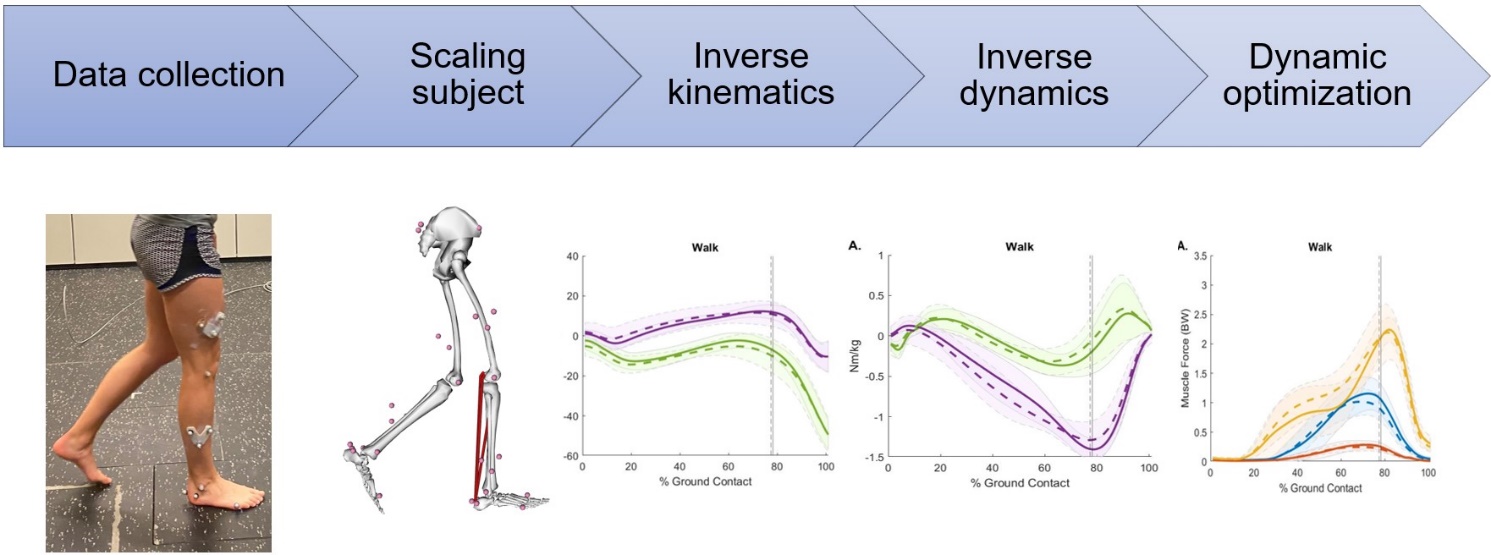


**Supplementary Figure 2**. Overview of the experimental and modeling workflow in OpenSim from data collection to inverse kinematics and dynamics, to result in simulated triceps surae muscle forces.


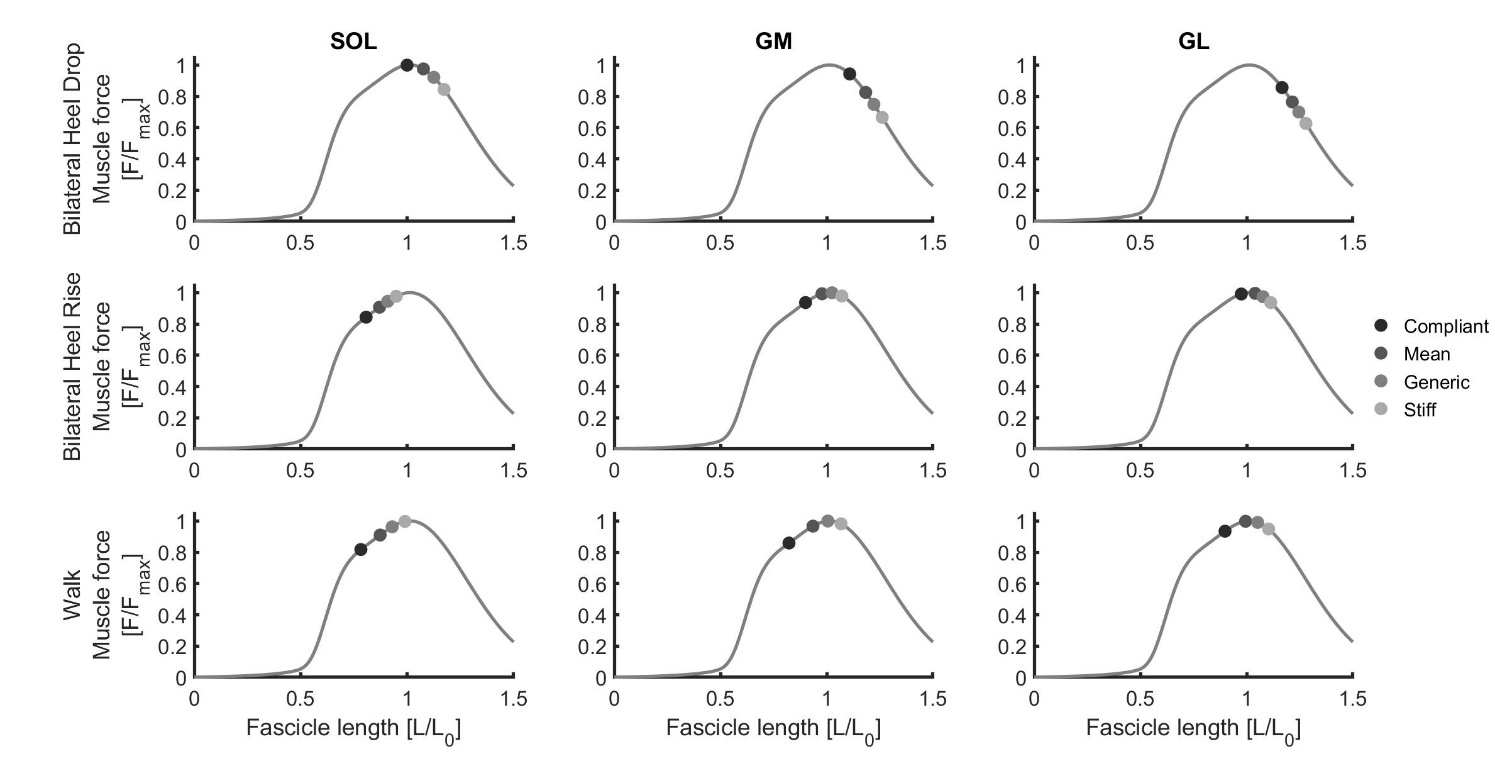


**Supplementary Figure 3**. Force-length relationship of the SOL, GM, and GL muscles for the different compliance models (compliant (black), mean (dark grey), generic (concrete), and stiff (light grey)) for the bilateral heel drop (top row), bilateral heel rise (middle row), and the walking (bottom row) exercise at the moment of peak triceps surae force.


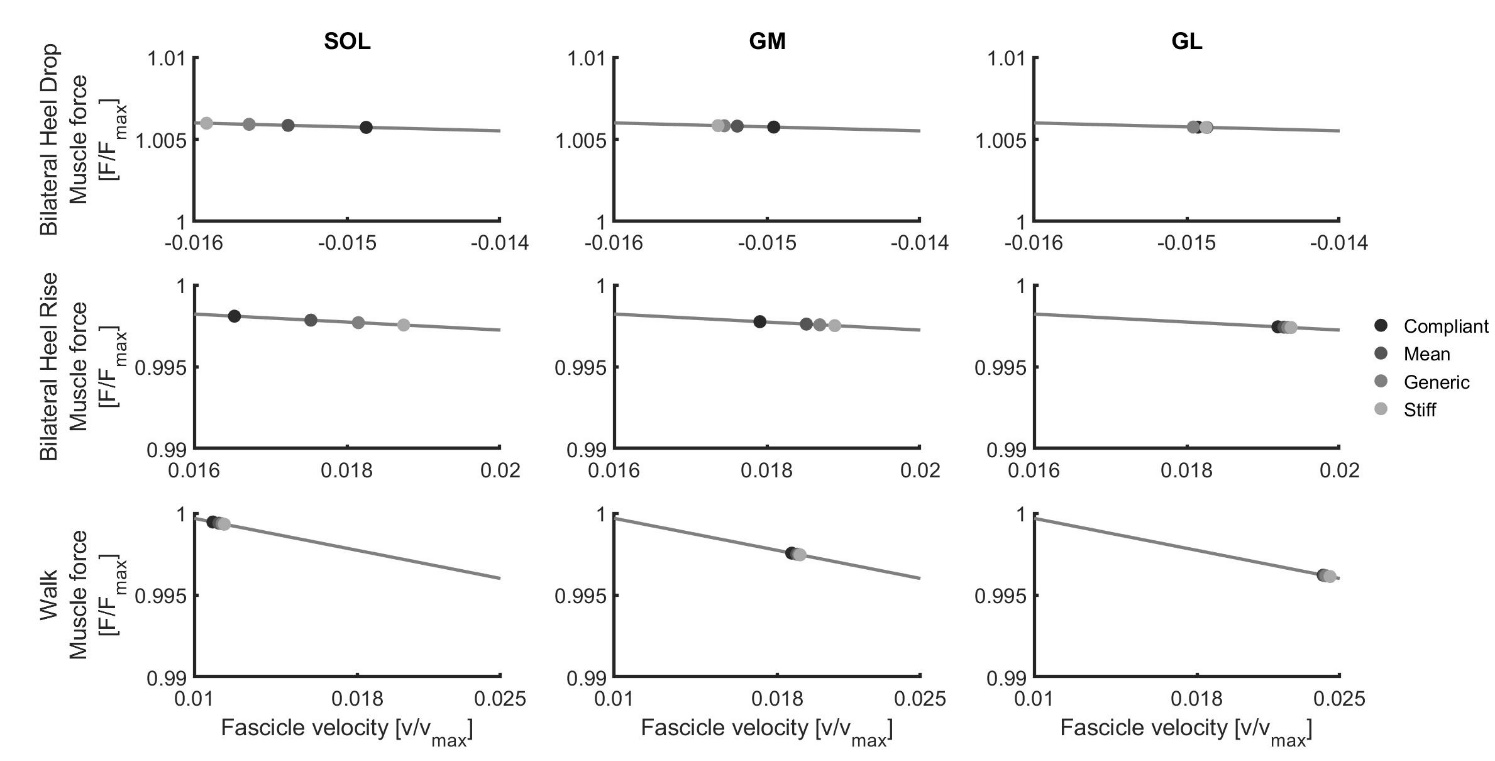


**Supplementary Figure 4**. Force-velocity relationship for the compliant (black), mean (dark grey), generic (concrete), and stiff (light grey) models for the three different exercises (top row: bilateral heel drop; middle row: bilateral heel rise; bottom row: walk) and the three muscles (left column: soleus (SOL); middle column: gastrocnemius medialis (GM); right column: gastrocnemius lateralis (GL)).
